# Efficacy of vitamin D_2_ in maintaining serum total vitamin D concentrations and bone mineralisation in adult dogs fed a plant-based (vegan) diet in a 3-month randomised trial

**DOI:** 10.1017/S0007114523001952

**Published:** 2024-02-14

**Authors:** Sarah A. S. Dodd, Jennifer Adolphe, Cate Dewey, Deep Khosa, Sarah K. Abood, Adronie Verbrugghe

**Affiliations:** 1 Department of Population Medicine, Ontario Veterinary College, University of Guelph, Guelph, Canada; 2 Department of Veterinary Biomedical Sciences, University of Saskatchewan, Saskatoon, Canada; 3 Department of Clinical Studies, Ontario Veterinary College, University of Guelph, Guelph, N1G 2W1, Canada

**Keywords:** Body composition, Canine nutrition, Cholecalciferol, Dual-energy X-ray absorptiometry, Ergocalciferol

## Abstract

Dogs are considered omnivores based on their evolution consuming diets including animal tissue. Few feeding trials evaluating the nutritional suitability of exclusively plant-based (vegan) diets in dogs have been published, and the efficacy of vitamin D_2_ in maintaining canine serum vitamin D levels has not been clearly determined. A blinded dietary trial included sixty-one healthy desexed adult dogs: thirty-one fed an experimental extruded vegan diet (PLANT) and thirty fed a commercial extruded meat-based diet (MEAT) for 3 months. Dogs were screened via veterinary examination and routine laboratory analyses prior to enrolment, at baseline and exit timepoints. Body composition was measured by dual-energy X-ray absorptiometry and blood was collected for vitamin D profiling. All dogs maintained health parameters, body weight and composition throughout the study. Dogs maintained on PLANT demonstrated a significant reduction in platelet count, creatinine, blood urea nitrogen and cholesterol, though values remained within normal reference ranges. Dogs fed PLANT also demonstrated a shift from vitamin D_3_ to vitamin D_2_ metabolites, though total vitamin D analogue levels were unchanged, with the exception of 24,25-dihydroxyvitamin D. Bone mineral content and density did not differ from baseline values. Health status was maintained in dogs fed PLANT and vitamin D_2_ appeared efficacious in maintaining serum total vitamin D concentrations and bone mineralisation. Findings support the hypothesis that PLANT was comparable to MEAT for maintenance of healthy adult dogs for at least 3 months and identified areas where further research is warranted to elucidate the potential risks and benefits of plant-based (vegan) diets.

The modern domestic dog, *Canis lupus familiaris*, descends from wolves (*Canis lupus*) but deviates from their wild-type ancestor as a result of co-adaptation with humans^(1)^. Both dogs and wolves fall within the family Canidae, order Carnivora^(2)^, and are considered omnivores or facultative carnivores based on their feeding strategies and gastrointestinal anatomy and physiology^(3)^. Though the diets of free-roaming wolves and dogs are characterised by intake of prey^(4)^, the domestic dog adapted to consume diet richer in plant materials^(5)^ and shows some preference for starch as a dietary energy source^(6)^. Most commercial dog foods contain animal ingredients; indeed, the pet food industry relies heavily on by-products from the slaughter of animals for human consumption^(7,8)^. Homemade foods for dogs also typically have a high inclusion of animal-derived ingredients, due to the common perception that dogs require meat as a main component of their diet^(9,10)^. In contrast, some dog owners elect to feed their dog a diet entirely devoid of animal ingredients^(11)^. These exclusively plant-based diets are often also termed ‘vegan’^(12)^. The widely accepted term ‘plant-based’ generally refers to a diet that does not include animal-derived ingredients, though it is not universally defined and may be used by some to refer to entirely animal-free ‘vegan’ type diets, while others may use it for diets that minimise, but do not completely exclude, animal products^(13–16)^. For the purposes of this article, the term ‘plant-based’ will be used in reference to an entirely animal-free ‘vegan’ type diet.

In North America, the industry guidelines for pet food formulation are based on profiles of indispensable nutrients, not ingredients^(17)^, and commercial vegan diets have been produced that meet all of these nutrient recommendations without the inclusion of any animal-derived ingredients^(18)^. Provision of some key essential nutrients, however, has proven challenging in diets that do not contain animal-derived ingredients, as methionine, methionine + cystine, taurine, Ca, phosphorus and vitamin D content have frequently been reported below industry recommendations in commercial vegan pet foods^(18–20)^. Given these findings, it would be expected that dogs fed vegan diets may be at increased risk of nutritional deficiencies and clinical manifestations of irregularities in body composition, Ca, phosphorus and vitamin D metabolism.

A dearth of knowledge exists on the link between animal-free diets and canine health. Previous studies have demonstrated a lack of effect on physical examination, haematological and serum biochemical characteristics^(21,22)^ and/or some additional serum nutrient evaluations^(22)^. Impacts on digestibility, faecal microbiota and glycaemic and insulinaemic responses have also recently been investigated in dogs^(23–25)^. To the authors’ knowledge, body composition changes in response to a vegan diet have not been investigated. Given the differences in amino acid provision from plant- or animal-derived protein sources^(26–28)^, it is possible that increased mobilisation of amino acids from body stores could evoke changes in lean soft tissue (LST) mass^(29)^. Furthermore, while suitable non-animal-derived dietary sources of Ca and phosphorus are plentiful^(17)^, bone metabolism in dogs fed exclusively plant-based diets could be affected by the source of dietary vitamin D^(30)^. In most mammals, vitamin D can be produced in the skin with sufficient UV light exposure, though it has been demonstrated that endogenous synthesis is minimal in dogs, rendering dietary fortification essential for this species^(31–33)^. Dietary vitamin D can be provided as two different compounds: cholecalciferol (vitamin D_3_), predominantly derived from animal sources, or ergocalciferol (vitamin D_2_) derived from non-animal sources^(32)^. Though non-animal sources of vitamin D_3_ exist^(34,35)^, these ingredients have yet to be accepted into pet food manufacturing. In cats, dietary vitamin D_2_ was less effective compared with vitamin D_3_ in maintaining total serum vitamin D^(36)^. Based on existing research, the efficacy of D_2_ in maintaining serum vitamin D has not been well established in dogs, and industry recommendations for vitamin D fortification of pet food are based on studies determining adequate levels of vitamin D_3_ to avoid signs of deficiency or toxicity^(32,33)^. If vitamin D_2_ intake results in abnormal vitamin D metabolism in dogs, this could impact bone mineral content and bone mineral density^(37)^.

The objectives of this study were thus to compare general health markers, body composition, bone mineralisation and serum markers of vitamin D metabolism in dogs maintained on either an exclusively plant-based ‘vegan’ diet or a conventional-type canine diet inclusive of animal products. It was hypothesised that body composition, bone mineralisation and serum markers of vitamin D metabolism would not be affected by diet and that a nutritionally adequate extruded vegan diet would be comparable to a similar diet containing meat-based ingredients with respect to maintenance of healthy adult dogs.

## Methodology

This study was conducted at the University of Guelph with the approval of the Research Ethics Board (Research Ethics Approval number 19-02-036) and Animal Care Committee (Animal Use Protocol #4129), ensuring the research protocol was in line with institutional, provincial and national guidelines and policies for humans participating in research as well as for the care and use of animals in research. The sample size chosen for this study was based on previous vitamin D-related nutrition studies which have utilised a similar number of dogs^(38–43)^ and justified with sample size calculations based on previously reported average bone mineral density of dogs^(44)^. For sample size calculation, confidence was set at 0·95 and power to 0·8. The calculated minimum sample size per treatment group was 27 dogs with a 1 to 1 allocation ratio, with an expectation of 10 % dropout, resulting in a target sample size of thirty dogs per group.

### Participant enrolment

Recruitment for trial participants was initiated on 3 June 2019 and completed on 5 July 2020. The first author (SD) was responsible for recruitment and enrolling of all participants. An eSurvey was designed on the Qualtrics platform to collect data regarding patient suitability. This survey was promoted locally through printed advertisements around the university campus and surrounding community and shared virtually on social media to local dog-related groups. The first page of the questionnaire provided information on the study and served to provide informed consent for participation in the recruitment survey. Participants could only continue the questionnaire if they confirmed they understood their rights regarding participation in the survey and ability to exit the survey at any time. Potential participants were informed that questions were required to be answered honestly and sensitive personal details would be accessible to the research team. Participants were also provided with a description of the expectations of their participation, which included that they would bring their dogs for a total of three visits to the Ontario Veterinary College, would feed the study diet and follow the study protocol and consent to their dogs undergoing physical examinations, blood draws and dual-energy X-ray absorptiometry (DEXA). There were no incentives for study participation, though benefits included complimentary veterinary examination, blood analyses, urinalysis, DEXA measurement of body composition and provision of the study diet for 4 months – 1-month adaptation, followed by 3-month experimental period. Information collected included: dog age, sex, body weight (BW) and body condition score (BCS, evaluated by selection of WSAVA BCS image^(45)^ most closely resembling their dog, ordered randomly to attempt to avoid bias); the main diet fed; details of treats and snacks; provision of supplements and medications; number of adults in household; presence of children in the household and interaction between children and dog feeding; presence of other pets in the household and access to other pets’ food, dog housing (indoors *v*. outdoors); feeding management; access to unmonitored food sources; dog activity and dog medical history. Dogs were excluded from consideration if they were reproductively intact, weighed less than 5 kg, had an owner-reported BCS > 5, fed a homemade or raw diet, housed outdoors without supervision, had access to unmonitored food sources, had current medical problems, received medication other than parasite preventatives, had previous medical problems that could affect them currently (e.g. previously diabetic but in remission, recurrent ear infections, etc.) or had known dietary allergies. Dogs in households without children or other pets were prioritised for inclusion in the study as it was considered that the dietary intakes of dogs with potential access to other pets’ foods or unattended with children would potentially be less reliable than those living in households with adults only.

A total of 569 surveys were undertaken and 280 were completed in full. Of these, 132 survey participants met inclusion criteria and were selected for invitation into the study. An email was sent with more detailed information regarding participation in the study and invitation to attend an enrolment appointment. Replies were received from seventy-eight potential participants, with seventy-two enrolment appointments scheduled for eighty-seven dogs (some participants had more than one dog).

Enrolment appointments involved discussion of the study procedures, collection of a signed informed consent form for participation in the study and a wellness examination conducted by a veterinarian. In order to minimise variability, the first author (SD) assessed all dogs at all visits. The examination included collection of medical and dietary history, a physical examination, BW measured using a weigh scale and blood was collected for complete blood count (CBC) and serum biochemistry. Participating dog characteristics are included in online Supplementary Table 1.

### Diets

Two isoenergetic extruded diets, one commercial animal-inclusive diet^1^ (MEAT) and one purpose-formulated experimental exclusively plant-based diet (PLANT) were used; both met or exceeded nutrient recommendations for adult maintenance^(17)^. The MEAT diet was supplemented with vitamin D_3_, while the PLANT diet was supplemented with vitamin D_2_, representative of diets commercially available for dogs. The diets were packaged into identical sealed white bags and labelled. The investigators and participants were blinded to the identity of the diets. Diet identities were kept by a third person employed at the University of Guelph, who was not involved in data collection, statistical analysis and data interpretation, until statistical analyses were complete.

Nutrient analyses were performed on the diets post-manufacturing (online Supplementary Table 2). Proximate analyses (moisture, protein, fat, ash, crude fibre) and minerals were performed at a commercial laboratory (Bureau Veritas). Moisture, crude protein, crude fat, ash and minerals were measured by AOAC methodology (AOAC 992.15, 996.06, 923.03, 084.27, 935.29). Crude fibre was measured by AOCS Ba 6a-05 and iodine by neutron activation. Carbohydrate content, approximated as nitrogen-free extract (NFE), was calculated by the equation: NFE = 100 – crude protein – crude fat – crude fibre – ash)^(17)^. Metabolisable energy was calculated as energy content per kg as fed, using the equation: ME (kcal/kg) = 10[(3·5 × crude protein)+(8·5 × crude fat)+(3·5 × NFE)], with crude protein, fat and NFE values as g/100 g as fed^(17)^. Samples underwent hydrolysis, oxidised hydrolysis and alkaline hydrolysis before amino acid profiling were performed by ultra high performance liquid chromatography coupled with mass spectrometry^(46)^. Lastly, vitamins were measured at a commercial laboratory (DSM). Vitamins A and D were measured by AOAC methodology (AOAC 974.29.45.102 mod and 2011.12 (modified), respectively), and B vitamins were measured by QDa^(47)^, with the exception of biotin while cobalamin was outsourced to a second commercial laboratory (Covance).

Food quantity was calculated based on the dog’s current dietary intake to match energy content and maintain current BW, and a gram scale was provided to each household to precisely measure out the recommended quantity of food per day. Participants were instructed not to feed their dogs any other food for the duration of the study, with exceptions for allowance for treats. Participants were given a list of treats that could be fed for the duration of the study (entirely plant-based treats without added micronutrients, fruits and vegetables); an acceptable treat dose was given for each dog to avoid exceeding 10 % of their daily energy intake from sources other than the trial diet.

### Diet trial

Originally, the diet trial was scheduled to be completed before the end of September 2020. However, as a result of the global COVID-19 pandemic, the University of Guelph closed down for research and the diet trial was paused in March 2020, until research was allowed to resume in July 2020 with implementation of appropriate public health protocols. During the lockdown, dogs participating in the trial were maintained on the diet, either MEAT or PLANT depending on their phase of the study (adaptation or experimental period) and experimental group, in order to allow immediate resumption of data collection when the facilities were reopened. This resulted in variability in the duration of dogs participating in the trial, with seven dogs consuming the baseline diet for more than 4 weeks (three in group PLANT and four in group MEAT), and three dogs consuming the experimental diet (three in group PLANT) for more than 12 weeks.

Online Supplementary Fig. 1 depicts the timeline of the trial. An initial adaptation period of 4 weeks was performed between the screening and baseline appointments to ensure all dogs started the trial from the same diet and mitigate variation due to differences in pre-trial diets. Although all dogs enrolled in the study were fed commercial diets prior to their participation, there was variation of ingredients and nutrient profiles of these diets, including six dogs previously fed commercial vegan diets. During the adaptation period, all dogs were fed MEAT. Upon completion of the 4-week adaptation phase, the dogs were scheduled baseline appointments and randomly allocated to treatment groups (MEAT or PLANT) prior to the baseline evaluation, using an online random number generator (Google Random Number Generator, Google LLC), with even numbers being allocated to PLANT and odd numbers allocated to MEAT. Investigators were blinded to the identity of either group; they were encoded as group 1 and 2. Baseline evaluations consisted of a veterinary wellness examination (medical and dietary history, physical examination, CBC, serum biochemistry and urinalysis), body composition analysis by DEXA and blood collection for vitamin D profile. BW, BCS, CBC, serum biochemistry and urinalysis were analysed as measures of general health and wellness, as typically performed for routine veterinary wellness examinations^(48)^. Participants were advised to fast their dogs for 12 h prior to the appointment.

After the baseline evaluation, dogs were transitioned to their respective experimental diet – either continuing on MEAT or starting PLANT. Dogs were fed the trial diets for 12 weeks and then returned at the end of the trial for their exit evaluation. The study duration of 12 weeks was decided upon as a balance between ability to detect effects, should there be differences between the diets, and owner compliance over an extended time period. Based on the rate of canine detectable signs of deficiency in response to alterations in dietary vitamin D, 12 weeks was considered a sufficient time period to be able to detect differences between groups, without risking the health of individual dogs should vitamin D_2_ prove to be an unsuitable source of dietary vitamin D for adult maintenance^(49–52)^. Exit evaluations were the same as the baseline evaluations. Throughout the study, including the 4-week adaptation and the 12-week experimental period, participants were asked to record quantity of food offered, quantity of food eaten, amount and type of snacks or treats provided, frequency of defaecation, faecal condition score, BCS, BW, duration of walks or play activity and any other notable events in a daily diary. Faecal condition score and BCS charts were provided^(45,53)^.

### Body composition analysis

Body composition was measured by DEXA (Prodigy Advance, GE Healthcare). Dogs were placed in ventral recumbency and positioned with forelimbs extending caudally, adjacent to but not touching the body wall, and hindlimbs extending caudally away from the body, as previously described^(54)^. Total body scanning mode was utilised within the scanner’s software (enCORE Version 16, GE Healthcare), with the software selecting thick, standard or thin settings according to the dog’s BW. Body fat mass (BF), LST mass, bone mineral content and total tissue mass were measured in g, from which proportions of body fat (BF %), lean soft tissue (LST %) and bone (Bone %) were calculated. Proportions were calculated as tissue mass divided by total tissue mass, multiplied by 100 %. Bone mineral density was measured as radiation energy per pixel converted into area density, measured in g/cm^2(55)^. Dogs were scanned in duplicate and values from the two scans were averaged.

For determination of body composition, dogs were sedated with dexmedetomidine (2–10 µg/kg BW; Dexdomitor®, Zoetis) and butorphanol (0·2 mg/kg BW; Torbugesic®, Zoetis) administered together in the same syringe, intravenously via the cephalic or lateral saphenous vein or intramuscularly in epaxial muscle, depending on the behaviour of the dog. If a top-up of sedation was required during the procedure, only dexmedetomidine dose was repeated and administered intravenously, starting at 50 % of the initial delivered dose. Upon completion of the body composition analysis, sedation was reversed with atipamezole (0·02–0·1 mg/kg intramuscularly; Antisedan, Zoetis), administered intramuscularly in the epaxial muscles in a volume matching the administered dexmedetomidine volume. During sedation, heart rate, respiratory rate and temperature were monitored in each dog prior to and immediately after administering sedation, prior to and after moving and positioning dogs on the DEXA table, prior to, in between scans and after completion of DEXA scanning and prior to and after administering the sedation reversal until the dog recovered. Recovery was established once dogs were able to stand and ambulate unassisted. Sedation doses were recorded, and the same dosing as administered at the exit appointment was administered at the baseline appointment, as suitable.

### Blood and urine collection

Blood collections were performed at screening, baseline and exit timepoints, while urine collection was performed at baseline and exit timepoints only.

At the screening evaluation, 6 ml of blood was collected by venipuncture of the saphenous or cephalic vein using a 22G BD Vacutainer® needle into a 3 ml BD lavender-top EDTA Vacutainer® tube and a 3 ml BD red top plain Vacutainer® tube. At the baseline and exit timepoints, blood was collected while the dog was sedated for DEXA scan. Once reversal (atipamezole) was administered, dogs were positioned in lateral recumbency for blood collection. Venipuncture was performed via the saphenous, or jugular vein, depending on dog size, using a 22G BD Vacutainer® needle (Becton, Dickinson and Company). Fifteen millilitre of blood was collected into five 3-ml BD red top Vacutainer® blood collection tubes with no additives and 6 ml was collected into two 3-ml BD lavender top EDTA Vacutainer® blood collection tubes. After clotting for 30 min at room temperature, serum was separated from whole blood in plain red top tubes by centrifugation at 1,500 ***g*** in a refrigerated centrifuge set to 4°C. Samples for same-day testing, including samples for CBC and serum biochemistry, were submitted immediately for analysis. Remaining sera aliquots were immediately frozen at −80°C in 1·5 ml Eppendorf tubes and stored until analysed.

At baseline and exit timepoints, dogs were walked prior to their examination and sedation, and free-catch urine collected into plain 12 ml tubes. In a small number of dogs for which free-catch urine collection was not possible prior to DEXA scan, urine was collected via ultrasound-guided (Clarius C3, 2–6 MHz frequency; Clarius) cystocentesis performed after completion of the scans. Urine collection method was recorded for each dog. Urine samples were submitted immediately for analysis.

### Complete blood count, serum biochemistry and urinalysis

At each timepoint, CBC, serum biochemistry and urinalyses were performed at the Animal Health Lab of the Ontario Veterinary College, University of Guelph (Guelph, Ontario, Canada) on the same day as sample collection. CBC with machine differential was performed on whole blood mixed with EDTA using a Siemens ADVIA 2120 haematology analyser (Siemens AG). Photometric serum biochemistry was analysed by Roche Cobas 6000 c501 (Roche Holding AG). Urinalysis was performed by Siemens Multistix urine dipstick read on the Siemens Clinitek Plus urine strip reader (Siemens AG), with a microscopic examination of the urine sediment by an experienced laboratory technician.

### Vitamin D and analogues

Frozen sera for baseline and exit samples were shipped on dry ice to external analytical laboratories after trial completion. Vitamin D profile, including 25-hydroxyvitamin D, parathyroid hormone (PTH) and ionised calcium (iCa), was performed by the Veterinary Diagnostics Laboratory of the Michigan State University (Lansing, Michigan, USA). Analysis of 25-hydroxyvitamin D and PTH was performed by commercial RIA (Diasorin; Scantibodies), while iCa was measured with an ion-sensitive electrode (NOVA 8 bioanalyser, Nova Medical).

Serum vitamin D analogues were analysed by Heartland Assays. Vitamin D_2_ (25(OH)D_2_), vitamin D_3_ (25(OH)D_3_), calcitriol (1,25(OH)_2_D_2_, 1,25(OH)_2_D_3_), 24,25(OH)_2_D_2_ and 24,25(OH)_2_D_3_ were measured by liquid chromatography tandem mass spectrometry (LC/MS/MS). This laboratory participates in the Vitamin D External Quality Assessment Scheme^(56)^, as well as the Centers for Disease Control and Prevention Vitamin D Standardisation-Certification Program^(57)^. Vitamins D_2_ and D_3_ as well as 24,25(OH)_2_D_2_ and 24,25(OH)_2_D_3_ were extracted and quantified by the same method. Serum samples along with standard curve and controls were precipitated with 0·2M ZnSO_4_ and then vortexed. Methanol was added and all samples were vortexed again. Then, D_2_ and D_3_ internal standards were added and vortexed. Hexanes were added to all samples and controls and then vortexed, followed by centrifugation. The organic layer was transferred and dried, and then all standards, controls and samples were reconstituted with LCMS grade methanol and water containing 0·1 % formic acid, before loading onto the auto-sampler for LC/MS/MS analysis (Agilent 1290 infinity HPLC coupled to Agilent 6400 MS/MS with ESI source).

For extraction and quantification of 1,25(OH)_2_D, samples, controls and standards were first spiked with d6-1,25(OH)_2_D_3_ internal standard before protein precipitation. Samples and controls were purified by liquid–liquid extraction, followed by solid phase extraction. Samples, controls and standards were then derivatised using 4-phenyl-1,2,4-triazoline-3,5-dione, followed by a second solid phase extraction. Samples and controls were reconstituted in LC/MS/MS mobile phase before injection and analysis (Agilent 1290 HPLC system using a Zorbax Eclipse Plus C-18 column coupled to 6460 Agilent triple tandem MS/MS in positive ion mode using an ESI source). Inter-assay variation for 25(OH)D and 24,25(OH)_2_D was reported at less than 5 %, while inter-assay variation for 1,25(OH)_2_D was 9·4 %.

### Statistical analyses

All analyses were performed using commercial statistical software (StataIC, StataCorp.). Independent variables (dog sex, age, BW, BCS and season) were tested for normality of distribution using Shapiro–Wilk normality test and visual evaluation of frequency histograms and normal probability plots. Differences in distribution of independent variables (age, sex, BCS) between diet groups after randomisation were tested using *t* test for parametric and Wilcoxon rank-sum test for non-parametric data. Dependent variables (CBC, biochemistry, urinalysis, body composition, vitamin D metabolites) were tested for normality of distribution using Shapiro–Wilk normality test and visual evaluation of frequency histograms and normal probability plots. Non-parametric data were log transformed prior to analyses (blood cell counts, MCV, MPC, plateletcrit, CO_2_, albumin, globulin, albumin:globulin, urea, glucose, conjugated bilirubin, ALP, steroid-induced ALP, GGT, ALT, CK, amylase, lipase, osmolality, all urinalysis variables, 25(OH)D_2_, 25(OH)D_3_, total 25(OH)D, 1,25(OH)_2_D_3_, 24,25(OH)_2_D_3_, total 24,25(OH)_2_D). Categorical data including urine colour and clarity were coded into ordinal scales from 0 to 3, with 0 being pale yellow or clear, respectively, and 3 being dark yellow or cloudy, respectively. Ordinal scales of 0–3 represented negative, trace and 1–3+ values reported by the laboratory for the presence of protein, glucose, ketones, bilirubin, blood, epithelial cells, crystals, fat and bacteria. Blood cell counts of none, rare and 1–100+ cells per high power field were translated into ordinal scales of 0–6. Ordinal data were treated as non-parametric.

Differences in dependent variables between dogs of different age, sex and BCS were tested using ANOVA and post-hoc Tukey test, as indicated. Univariate analyses were performed via repeated measures mixed model, using residual maximum likelihood, with time and treatment as fixed effects and dog ID as the variable on which data were repeated. As differences were detected between groups at baseline for some dependent variables (serum leucine, urine epithelial cells), baseline was included as a variable for all analyses. Post hoc analyses were performed by contrasting main effects and graphing the interactions. As multiple comparisons were made, the critical cut-off for *P*-values was adjusted to control for false discovery rate of less than or equal to 10 % according to the Benjamini Hochberg procedure as recommended for bioinformatics data^(58)^. Labelled scatter plots were created for each significantly different dependent variable to identify potential outliers.

## Results

### Group randomisation and trial completion

The independent variables considered for each group were sex, age, BW, BCS and the seasons during which the dog was participating in the study. After randomisation, the distribution of independent variables between groups did not differ (online Supplementary Table 3). During the adaptation phase, when all dogs were fed the MEAT diet, five dogs were dropped from the study as they did not like or tolerate the food – one dog refused to consume it, two showed signs consistent with adverse food reaction, with one dog producing loose faeces while the other exhibited episodes of bilious vomiting, and two dogs developed anal gland complications. A further four dogs were discontinued from the study during the adaptation phase as a result of the COVID-19 forced lockdown and when the research project resumed the participants declined to continue. Of the sixty-seven dogs completing the adaptation phase, sixty-six underwent baseline evaluations. One dog was excluded due to excessive weight gain during the adaptation period resulting in obese habitus. Of the sixty-six dogs completing baseline evaluations, sixty-one completed the trial and underwent exit evaluations. Three dogs were lost to follow-up during the trial, one specifically as a result of the COVID-related delays. One dog developed a urinary tract infection, was managed medically including prescription of a therapeutic diet and was discontinued from the study. Another dog developed gastric ulceration after administration of a non-steroidal anti-inflammatory, underwent medical management and was discontinued from the study. Of the sixty-one dogs completing the trial, three experienced delays in scheduling the exit appointment due to the COVID-19 pandemic, all three were in the PLANT group. Two dogs were maintained on the experimental diet for an additional 4 weeks (total 16 weeks) and the other for an additional 24 weeks (total 36 weeks). Examination of labelled scatter plots revealed these dogs to follow the same trends as the main body of the group, none were identified as outliers or had results significantly different from the dogs consuming the experimental diet for the intended 12 weeks.

### General health and wellness

Initially, only dogs confirmed by veterinary examination to have a BCS of 4–5 out of 9 were enrolled in the study, but due to a lack of healthy-weight dogs, allowance was made for overweight (BCS 6–7 out of 9) but not obese (BCS 8–9 out of 9) dog participation. BW and BCS were consistent between groups and between timepoints. Body fat as measured by DEXA was in agreement with veterinary assessment of BCS, based on reported values of body fat for each score in the system used^(59)^. There were no differences in CBC or biochemistry variables between groups after baseline.

On CBC, dogs fed MEAT demonstrated a significant increase in red cell parameters, including red cell count (erythrocyte), Hb and haematocrit, while dogs fed PLANT showed a decrease in platelet count and corresponding increase in mean platelet volume (Table 1). Serum biochemistry revealed significant differences in electrolytes, urea, creatinine, cholesterol and osmolality (Table 1). All CBC and biochemical values remained within the laboratory reference ranges.^2^

**Table 1.** Significant results of repeated mixed model analysis of complete blood count and serum biochemistry between dogs fed a plant- (PLANT, *n* 31) or animal-based (MEAT, *n* 30) diet for 3 months (95 % confidence intervals)

Analyte	Diet	Contrast	95 % CI	*P*	Reference range and units
Erythrocyte	MEAT	0·17	0·053, 0·280	0·004	5·8–8·5 × 10^12/l
Hb	MEAT	4·69	2·150, 7·231	<0·001	133–197 g/l
Haematocrit	MEAT	0·01	0·005, 0·020	0·001	0·39–0·56 l/l
Platelets	PLANT	−31·69	−13·114, −50·269	0·001	117–418 × 10^9/l
Mean platelet volume	PLANT	2·13	1·281, 2·972	<0·001	7–14 fl
Ca	PLANT	−0·05	0·082, −0·022	0·001	2·50–3·00 mmol/l
Mg	PLANT	0·38	0·016, 0·060	0·001	0·7–1·0 mmol/l
Urea	PLANT	−1·09	−1·651, −0·523	<0·001	3·5–9·0 mmol/l
Creatinine	PLANT	−17·87	−22·524, −13·209	<0·001	20–150 µmol/l
Cholesterol	PLANT	−1·12	−1·446, −0·797	<0·001	3·60–10·20 mmol/l
Osmolality	MEAT	2·53	0·790, 4·272	0·004	mmol/l

Urine transitional epithelial cells, fatty casts, fine and coarse granular casts were too infrequent for statistical analysis. At the exit timepoint, epithelial cells were significantly lower in the urine of dogs fed PLANT than dogs fed MEAT (-0·69 cells per high power field, 95 %CI −1·083, −0·284, *P* < 0·001).

### Body composition

**Fig. 1.**
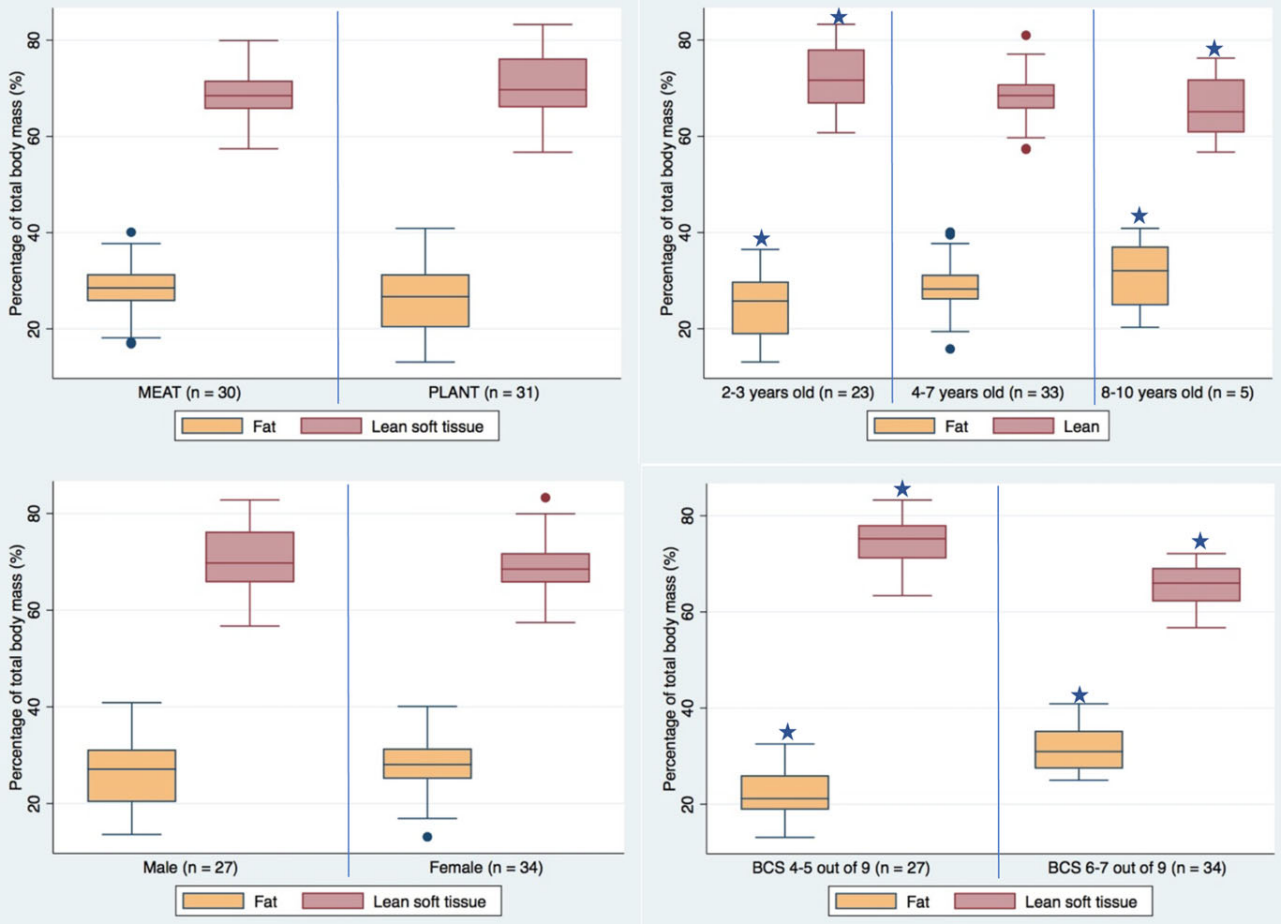
Dual energy X-ray body composition assessment in dogs fed a plant- (PLANT, *n* 31) or animal-based (MEAT, *n* 30) diet at the end of the 3-month diet trial. Stars indicate significant differences (*P* < 0·05) in body composition between categories as detected by one-way ANOVA.

Body composition was not affected by sex but differed according to age and BCS (Fig. 1), as BF % increased and LST % decreased with age and overweight body condition. However, as distribution of dependent variables did not differ between the two groups (online Supplementary Table 3), further control was not considered necessary. Body composition did not differ between diet groups or within groups over time (Fig. 1).

### Vitamin D metabolites and bone mineralisation

**Fig. 2.**
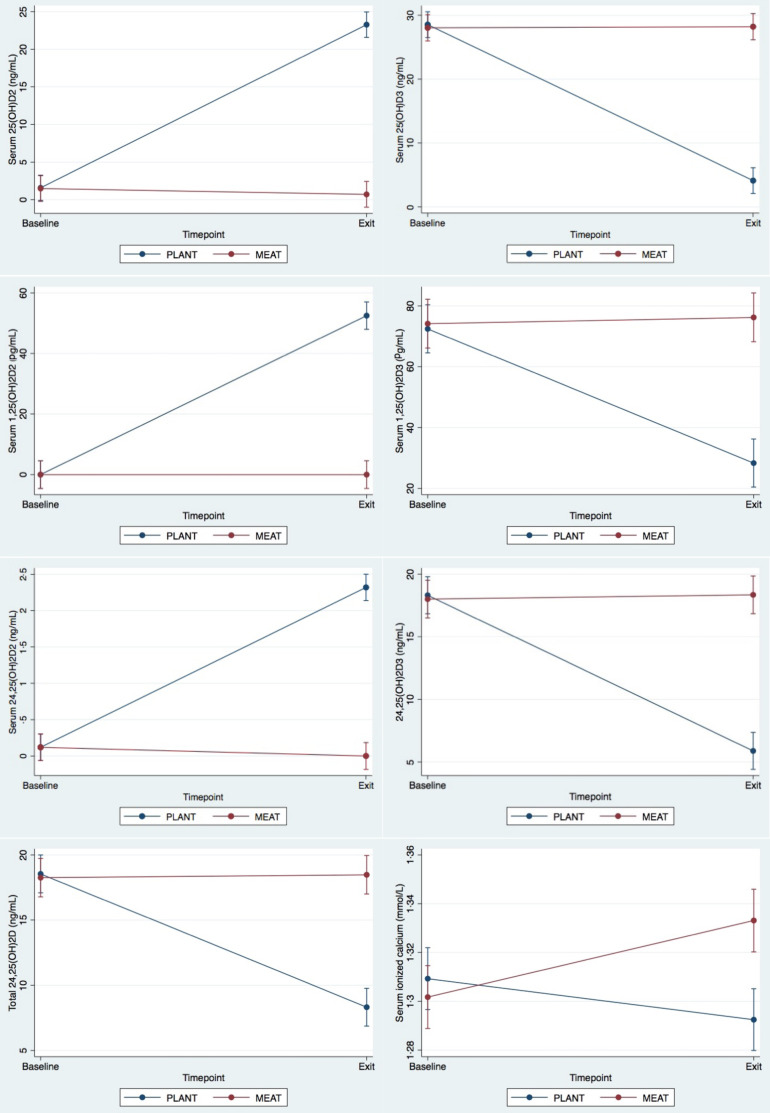
Significant differences in vitamin D metabolites between dogs fed PLANT (*n* 31) or MEAT (*n* 31) diet for 3 months.

At the exit timepoint, differences between groups were detected in serum concentrations of 25(OH)D_2_, 25(OH)D_3_, 1,25(OH)_2_D_2_, 1,25(OH)_2_D_3_, 24,25(OH)_2_D_2_, 24,25(OH)_2_D_3_, total 24,25(OH)_2_D and iCa (Fig. 2). In the PLANT group, all serum D_3_ metabolites decreased, and all serum D_2_ metabolites increased. As well, total serum 24,25(OH)_2_D concentrations decreased in the PLANT group. The only change in the MEAT groups was a slight increase in serum iCa concentrations. Despite the differences in vitamin D metabolism, there were no differences in Bone %, bone mineral content or bone mineral density between diet groups at exit or within groups over time.

## Discussion

The current study assessed the suitability of an extruded exclusively plant-based dry food formulated for adult maintenance in adult client-owned dogs compared with a commercial extruded diet containing animal-derived ingredients. The results support the hypothesis that a complete and balanced entirely plant-based diet is able to support general health and wellness in dogs, with no apparent adverse effects due to diet over a 3-month period. Throughout the trial, dogs maintained BW, BCS and body composition parameters in both diet groups. The results align with those reported by Wakshlag *et al.*, which demonstrated no differences in LST, BF or total BW in female dogs fed a high protein diet rich in either plant or animal protein^(29)^. Indices of health were within normal limits after feeding PLANT or MEAT for 3 months. Urinalysis did not appear to be impacted by diet in the manner expected in this study. Though it has previously been hypothesised that a vegan diet could result in increased urinary pH and predispose to formation of struvite crystalluria and urinary tract infection^(60)^, urine pH and quantity of struvite crystals were no different between dogs fed PLANT or MEAT, and the only observed effect of diet on urinary tract health was the presence of epithelial cells in the urine was lower in the dogs fed PLANT.

The main focus of this study was the use of vitamin D_2_ in place of vitamin D_3_ in its ability to maintain serum vitamin D and its analogues in dogs. Much of the vitamin D research that has been undertaken in this species has investigated vitamin D_3_ specifically. Keystone work in this area was published decades ago, including investigations into (lack of) cutaneous vitamin D synthesis^(31)^, effects of varying dietary levels of vitamin D_3_, Ca and phosphorus, predominantly on skeletal structure and development^(40,61–63)^, and reports describing signs of deficiency^(64)^, all leading to the current recommendations for vitamin D_3_ inclusion in canine diets^(32)^. More recent studies, case reports and reviews have reinforced these previous findings and advanced understanding of vitamin D_3_ metabolism and associated disorders in dogs^(65–74)^, but few studies have investigated the effects of dietary vitamin D_2_ in dogs and industry recommendations do not exist for this form of the vitamin.

A short review of the fate of dietary vitamin D is warranted prior to discussion of differential metabolism of vitamin D_2_ or D_3_. In short, ingested vitamin D is absorbed from the gut, bound to vitamin D binding protein (DBP) and transported in the blood predominantly to the liver, where it undergoes a first hydroxylation by cytochrome P450 enzymes, predominantly CYP2R1 and CYP27A1^(30,75,76)^. While vitamin D_3_ is capable of being 25-hydroxylated by either enzyme, vitamin D_2_ appears to be instead 24-hydroxylated by CYP27A1, with CYP2R1 responsible as the main 25-hydroxylase^(77)^. The resultant inactive mono-hydroxylated metabolite, 25(OH)D, is transported again bound to DBP to the kidneys, where it may interact with either one of two more cytochrome P450 enzymes: 1α-hydroxylation by CYP27B1 yields the most active vitamin D metabolite 1,25(OH)_2_D, known as calcitriol, or else CYP24A1 yields 24,25(OH)_2_D^(30,75,76)^. CYP24A1 is also capable of 24-hydroxylation of 1,25(OH)_2_D to form 1,24,25(OH)_3_D, considered the first step in a degradative pathway for vitamin D excretion^(75)^. This description is a simplification, as there are at least six enzymes with 25-hydroxylase activities^(76)^, present in the liver and extra-hepatic tissues, as well as extra-renal CYP27B1^(30,75,76)^, and multiple other vitamin D catabolites^(76)^, investigation of which was outside the scope of this project.

In other species, research has demonstrated differences in metabolism between vitamin D_2_ and D_3_. Mice and rats, omnivorous animals traditionally used as animal models in nutritional and medical studies, including those pertaining to vitamin D metabolism, appear to utilise vitamin D_2_ with equivalent efficacy as vitamin D_3_^(78)^.Yet in other omnivores, such as some primates, there is some discrepancy. Though a degree of discrimination against dietary vitamin D_2_ is common amongst non-human primates, this appears to be stronger in New World primates compared with Old World primates^(79,80)^. In humans, vitamin D_2_ was long considered to have equivalence to vitamin D_3_, though evidence has demonstrated otherwise^(81)^. Although oral doses of D_2_ or D_3_ have resulted in comparable reduction in circulating PTH, increase in vitamin D binding protein and free 25(OH)D in vitamin D deficient humans^(82,83)^, other studies have demonstrated greater efficacy of vitamin D_3_ in increasing serum 25(OH)D in non-deficient individuals^(84)^. The mechanism behind this may be associated with the specificity of DBP. In juvenile chickens (*Gallus gallus*), although oral vitamin D_2_ is capable of being converted to both 25(OH)D_2_ and 1,25(OH)_2_D_2_, the D_2_ metabolites bind poorly to DBP, resulting in little biological activity and rapid excretion^(85–87)^. In humans, it has been hypothesised that the greater efficacy of vitamin D_3_ in increasing serum 25(OH)D is also due to more rapid clearance of D_2_ and its metabolites due to the lower affinity of DBP for D_2_^(84)^. Discrimination against vitamin D_2_ has also been demonstrated in carnivorous mammals, such as domestic cats (*Felis catus)*. In cats, simultaneous oral dosing with D_2_ or D_3_ resulted in nearly double peak plasma concentrations of D_3_ than D_2_, while comparative inclusion in diets resulted in plasma concentrations of D_2_ around two-thirds of those of D_3_^(36)^.

Presently, the relationship between dietary vitamin D intake and vitamin D metabolism in dogs is poorly understood, with many complicating factors including dog age, breed, body condition and health status^(43,88,89)^. Furthermore, few canine studies have investigated vitamin D metabolites, most have focused exclusively on the biologically inactive, but easy to measure, 25(OH)D_3_. It is known that a dietary source of vitamin D for dogs is required because of negligible cutaneous synthesis^(31)^. Considering this dependency of dogs on diet as their vitamin D source, it was expected there would be significant differences in vitamin D metabolites between PLANT and MEAT-fed dogs, as the PLANT diet contained vitamin D_2_ exclusively while the MEAT diet contained vitamin D_3_ exclusively. Dietary levels of total vitamin D were slightly higher in the PLANT diet than the MEAT diet. In the dogs fed the PLANT diet, the most prevalent circulating vitamin D metabolite, 25(OH)D, was present predominantly as 25(OH)D_2_. This occurred despite being negligible at baseline, with 25(OH)D_2_ increasing to become the predominant metabolite by the exit timepoint, while, concurrently, 25(OH)D_3_ dropped. Comparatively, MEAT-fed dogs had negligible 25(OH)_2_D_2_ at both timepoints, with no change between baseline and exit. In both groups of dogs at exit, total 25(OH)D when calculated as the sum of 25(OH)D_2_ and 25(OH)D_3_ was comparable. Similar to 25(OH)D, the active vitamin D metabolite, 1,25(OH)_2_D was also present either as 1,25(OH)_2_D_2_ or 1,25(OH)_2_D_3_, depending on the dietary vitamin D source. In the dogs fed the PLANT diet, 1,25(OH)_2_D_2_ levels were almost double 1,25(OH)_2_D_3_ by the exit timepoint, with no changes in the MEAT-fed dogs. Total 1,25(OH)_2_D was comparable between groups. Most revealing, 24,25(OH)_2_D, the metabolite long considered to be the inactive waste metabolite of vitamin D, differed between the two groups. 24,25(OH)_2_D, while less potent than 1,25(OH)_2_D, plays a role in bone mineralisation, as well as being the precursor for 1,24,25(OH)_3_D, which is the inactive form excreted in the urine^(90,91)^. As with the other vitamin D metabolites, the D_2_ version, negligible in both groups at baseline, increased in the PLANT group, though 24,25(OH)_2_D_3_ remained more prominent. Total 24,25(OH)_2_D was reduced markedly in the dogs fed PLANT. The two triol metabolites, 1,25(OH)_2_D and 24,25(OH)_2_D, are both synthesised from calcidiol, either via 1-*α*-hydroxylase or 24-hydroxylase^(91,92,93)^. The decreased level of total 24,25(OH)_2_D may indicate an increased flux of 25(OH)D through to an unmeasured catabolite instead of 24,25(OH)_2_D, more rapid catabolism and/or excretion of 24,25(OH)_2_D and its catabolites and/or altered affinity of DBP for 24,25(OH)_2_D_2_. In both groups of dogs, no alterations in PTH were detected, though an increase in iCa was detected in the MEAT group. Considering that both groups were fed the MEAT diet for 4 weeks prior to the study, it was expected that the MEAT group would remain stable, and the increase in iCa was unexpected. There was no difference in total Ca in the MEAT group; thus, the increase in iCa may be attributed to a shift from bound Ca to iCa, not an increase in blood Ca levels altogether. Shifts from bound to free or iCa can occur with changes in blood pH, PTH or albumin levels^(94)^. As PTH and albumin levels were unchanged, it is possible that the increase in iCa demonstrated by the dogs fed the MEAT diet may have been attributable to a shift in blood pH, though acid-base balance was not evaluated in this study. In humans, a marginal increase in iCa has been reported for vitamin D deficient patients supplemented with D_3_ as compared with D_2_, leading to speculation that the two calciferols may have different effects on Ca homoeostasis outside of PTH, 25(OH)D or 1,25(OH)_2_D^(83)^. Previously, it has been demonstrated that 24,25(OH)_2_D_3_ and/or 1,24,25(OH)_3_D_3_ may have greater biological activity than 24,25(OH)_2_D_2_ or 1,24,25(OH)_3_D_2_, which may be a potential mechanism for the differences detected here. In contrast, iCa levels appeared to decrease in PLANT (Fig. 2), though the change did not attain statistical significance, while total serum Ca significantly decreased in this group. Ca provision in the PLANT diet was lower than the MEAT diet (online Supplementary Table 2) which may be one explanation for this variation, another could be attributed differences in 1,25(OH)_2_D in the PLANT group. Though total 1,25(OH)_2_D levels were not statistically different between PLANT and MEAT, the exit timepoint concentration of 1,25(OH)_2_D_2_ in PLANT was numerically lower than 1,25(OH)_2_D_3_ at the baseline timepoint (Fig. 2).

Bone mineralisation was comparable between both groups with no changes in bone mineral density, bone mineral content and total body bone %, with all parameters similar to those previously reported in healthy, young dogs^(95)^. Based on extrapolation from studies examining vitamin D and bone mineralisation in rats and humans, the 3-month duration was considered sufficient to detect changes in bone mineralisation, if vitamin D_2_ were ineffective as a dietary vitamin D source for dogs^(96,97)^. To the authors’ knowledge, these findings present the first published data in dogs to support dietary supplementation with vitamin D_2_ as a source of vitamin D, efficacious in maintaining active vitamin D metabolites and normal bone metabolism for at least 3 months. This could be used to update guidelines for pet food manufacturing, as presently industry recommendations have been generated exclusively from studies evaluating dietary vitamin D_3_^(32,33,98)^.

Though still within reference ranges, the observed changes in CBC and serum biochemistry could provide a direction for further research on possible benefits and adverse effects of meat-free and meat-inclusive diets in dogs. In comparison with baseline haematology, at the exit timepoint dogs fed PLANT had lower platelets counts but increased platelet volume. Dogs fed the MEAT diet demonstrated increased erythrocyte count, Hb and haematocrit. The changes in the MEAT group were particularly unexpected, considering the dogs were maintained on the same diet as they had been consuming when baseline haematology was performed. The lifespan of canine erythrocytes is around 115 d^(99)^, though changes in red cell count can be expected to be detectable within 2 weeks in response to alterations in haematopoiesis^(100)^.

The findings of increased platelet volume and lower platelet count in dogs fed the PLANT diet may be of interest. Adult men consuming no animal products demonstrated greater platelet volume, as compared with ovo-lacto vegetarian, moderate meat-eating or high meat-eating adult males^(101)^. In that study, mean platelet volume was associated with platelet phospholipid fatty acid profile, with larger platelets having higher linoleic acid and lower dihomo-*γ*-linolenic acid, EPA and docosapentaenoic acid concentrations. Potentially, differences in dietary fatty acid profiles between PLANT and MEAT could have affected platelet phospholipid fatty acid profiles. Although no adverse effects of the haematological changes were detected in the dogs fed PLANT, further research to determine the effect of entirely plant-based diets on platelet count, morphology and function in dogs is indicated.

Reduced serum cholesterol concentrations were also observed in dogs fed PLANT. By the end of the trial, differences were observed between groups though serum cholesterol was within the laboratory reference range^3^ for both diet groups. Though dietary cholesterol was not measured, this reduction of serum cholesterol concentrations may be explained by the lack of dietary cholesterol in PLANT, resulting in only endogenous cholesterol being measured. In comparison, dogs fed the MEAT diet had endogenous cholesterol supplemented with dietary cholesterol from the animal ingredients (primarily the chicken and egg ingredients) in the diet. Chicken tissues and eggs are known to contain high cholesterol content, consumption of which has been demonstrated to increase serum cholesterol concentrations in humans^(102–104)^. Lower serum cholesterol concentrations has been demonstrated in both men and women consuming no animal products, in comparison with ovo-lacto vegetarians, meat-eaters and fish-eaters^(105,106)^. In humans, hypercholesterolaemia is most commonly associated with atherosclerosis and subsequent CVD^(107–109)^. In dogs, although atherosclerosis can be experimentally induced, this is not a common naturally occurring condition^(110)^. Instead, hypercholesterolaemia occurs in and contributes to a number of other chronic disease states in dogs, including diabetes mellitus, hyperadrenocorticism, hypothyroidism, obesity, pancreatitis, primary/idiopathic hyperlipidaemia, primary hypercholesterolaemia and protein-losing nephropathy^(111–113)^. While there are no known benefits of dietary cholesterol restriction in healthy dogs, and no known health concerns for healthy dogs consuming cholesterol-containing diets, the findings of the present study suggest that a vegan diet could be beneficial as a therapeutic aid to reduce hypercholesterolaemia in dogs and investigation in hyperlipidaemic dogs may be warranted.

Other notable differences in serum biochemistry included reductions in creatinine and urea in dogs fed PLANT. Urea, measured as blood urea nitrogen (BUN), is a low-toxicity end product of protein metabolism and is eventually excreted in the urine^(114)^. As such, changes in BUN can be expected with changes in dietary protein level, with diets high in protein associated with higher BUN and diets deficient in protein reducing BUN^(115–117)^. The PLANT and MEAT diets, however, provided a similar quantity of dietary protein and were both in excess of the industry recommended minimum^(17)^. Though the trial diets were formulated to be iso-nitrogenous, testing of the completed product revealed a difference of 1·1 g/100 kcal between the two diets, with PLANT having the lower protein content. All other markers of protein metabolism, such as BW, body condition, LST, serum total protein and albumin concentrations, were maintained. Dogs fed PLANT showed no differences in indicators of energy metabolism or protein synthesis^(118)^, with serum glucose and protein levels, BW and LST all maintained and comparable to the MEAT-fed dogs. Furthermore, previous studies have demonstrated comparable digestibility of plant-based proteins in dogs fed plant- or chicken-based iso-nitrogenous diets^(119–122)^. Soya, a plant-based protein, has been reported to be beneficial for dogs with hepatic disease, as the amino acid profile may positively influence protein and amino acid metabolism and decrease blood ammonia levels^(122)^. It is thus unclear if the protein quantity or quality in PLANT contributed to the lower BUN in the dogs fed PLANT. In addition to protein metabolism, BUN is also a marker of renal function, along with serum creatinine, which also decreased significantly in dogs fed PLANT and was lower in these dogs than the dogs fed the MEAT diet. In both groups, BUN and creatinine were maintained within the normal laboratory reference range for the duration of the study. Creatinine can also be affected by muscle mass, as its precursor, phosphocreatine, is found in high concentrations in muscle^(123,124)^. However, all dogs in the trial maintained BW and LST; thus, the reduction in creatinine is unlikely to be associated with any changes in muscle mass. In humans, plant-based proteins and plant-based diets have been suggested to be associated with kidney health, with improved outcomes associated with hypertension, glomerular filtration rate decline and risk of developing chronic kidney disease^(125,126)^. Potentially, dogs fed PLANT experienced either decreased protein catabolism, as a result of lower dietary protein intake or reduced endogenous protein catabolism, and thus reduced BUN and creatinine synthesis and/or increased renal clearance of BUN and creatinine. Further research is required to determine if exclusively plant-based diets affect protein metabolism and renal function in dogs.

Interpretation of the data presented here must be performed with acknowledgement of the limitations of this study. The findings may not be applicable to vegan diets in general, as the diet used in this study was specifically formulated and tested to ensure nutritional adequacy as compared with the nutrient profile recommended by AAFCO for maintenance of adult dogs, but other vegan diets available commercially may not always do so^(18,20)^. Comparison was made only between an animal-based diet containing vitamin D_3_ (MEAT) and an animal-free diet containing vitamin D_2_ (PLANT). As it is convention within the industry to utilise vitamin D_3_ in most canine diets^(69)^, creating an experimental meat-based diet supplemented with vitamin D_2_ was not performed. Similarly, as animal-derived vitamin D_3_ would be considered unacceptable for inclusion in a commercial vegan diet, an experimental plant-based diet supplemented with vitamin D_3_ was not created. As such, this study design limits interpretation of the findings as to whether changes noted between groups were due to vitamin D source or the ingredient composition of each diet. However, considering the toxicity of vitamin D_3_ is 10–20 times greater than D_2_ in many other species^(32)^, there may be justification for inclusion of vitamin D_2_ as a supplement to otherwise animal-inclusive canine diets, conversely plant-based vitamin D_3_ may become available for use in pet food products in the future, so further investigation including D_2_ in an animal-based diet and D_3_ in an exclusively plant-based diet would be warranted. The stipulations of participation in the trial required feeding of the trial diets exclusively and limited treats and snacks, which may otherwise form a greater proportion of a dog’s daily food offering and could impact their nutrient intake. Given that privately owned dogs were enrolled in the study, the heterogeneity of the sample population could potentially be a source of confounding. However, based on the lack of differences found in the independent variables (sex, age, BW, BCS, season and breed) and general health and wellness indices (CBC, serum biochemistry) between groups at baseline, randomisation was considered successful with no predisposition to bias in either group. Another limitation presented by using privately owned animals was the duration of the trial. Using data extrapolated from other species or from other canine trials, the 3-month timeline was decided upon and was sufficient to demonstrate some differences between groups. However, it is possible that a longer trial could reveal further differences, or that variables would stabilise over a greater time period, though compliance with study protocols and retention time could become issues with longer study duration in client-owned dogs. Serial testing (i.e. testing at intervals and not just baseline and exit timepoints) would also provide more information about the changes discovered in some variables. Although 25(OH)D appears not to be affected by obesity in dogs^(89)^, it was nevertheless intended to only include dogs with an ideal BCS (4–5 out of 9^(45)^) in the study. However, many owners underestimated their overweight dogs’ BCS – a common finding regardless of the scoring system used^(127–129)^, and many overweight dogs were presented for participation. Lastly, the limitation of using only healthy, desexed dogs between the ages of 2–10 years of age limits extrapolation of these findings to a comparable proportion of the canine population. The aim of the study was to investigate the effect of vitamin D_2_ *v*. D_3_ on vitamin D metabolites and bone mineralisation in dogs, though vitamin D and its metabolites have many extra-skeletal roles as well^(30,68,88)^, though investigation of these was outside the scope of this study. More research is needed to further elucidate the health impact of exclusively plant-based diets and vitamin D_2_, particularly in growing dogs and senior animals.

### Conclusion

Bone mineralisation, serum vitamin D levels and iCa did not differ between groups, suggesting that vitamin D_2_ may be an efficacious dietary form of vitamin D for dogs. Research is warranted to further elucidate the effect of reduction in 24,25(OH)_2_D in dogs fed an entirely plant-based diet. For the duration of the study, all dogs maintained health and body composition. Although all health parameters were maintained within normal reference ranges, some differences were observed for dogs fed PLANT in comparison with dogs fed MEAT for some haematology and biochemistry biomarkers which provides direction for further research to elucidate the potential risks and benefits of vegan diets.

## Footnotes

1 Petcurean Go! Solutions Skin + Coat Care Chicken Recipe.

2 Animal Health Laboratory, University of Guelph.

3 Veterinary Diagnostic Laboratory, Michigan State University.

## Supplementary material

For supplementary material accompanying this paper visit https://doi.org/10.1017/S0007114523001952. click here to view supplementary material
