# Supplementary material for: Efficacy of vitamin D2 in maintaining serum total vitamin D concentrations and bone mineralisation in adult dogs fed a plant-based (vegan) diet in a 3-month randomised trial
Source: Br J Nutr. 2023 Sep 6;131(3):391–405. doi: 10.1017/S0007114523001952 (PMC10784131; doi:10.1017/S0007114523001952)
Supplement: Supplementary file 1 [file S0007114523001952sup001.docx]

**Supplementary Data**

**Table 1. Characteristics of the dogs enrolled to participate in the diet trial, with notes regarding drop-outs and losses.**

| Dog | Breed | Sex | Age (years) | Weight (kg) | BCS (1-9) | Group | Screening date | Baseline date | Exit date | Note |
| --- | --- | --- | --- | --- | --- | --- | --- | --- | --- | --- |
| 1 | Shih tzu | F | 5 | 6.9 | 6 | Excluded | July 2 2019 | DNF | DNF | Elevated liver enzymes |
| 2 | Shih tzu | F | 2 | 4.9 | 5 | Dropped | July 2 2019 | DNF | DNF | Did not like food |
| 3 | Labrador x | M | 6 | 36.5 | 7 | MEAT | July 2 2019 | Aug 1 2019 | Oct 22 2019 |  |
| 4 | Boxer x | F | 7 | 26.2 | 4 | PLANT | July 2 2019 | July 30 2019 | Oct 22 2019 |  |
| 5 | Labrador retriever | F | 7 | 28.3 | 6 | MEAT | July 4 2019 | July 30 2019 | Oct 22 2019 |  |
| 6 | Golden retriever | F | 4 | 28.3 | 6 | MEAT | July 4 2019 | Aug 1 2019 | Oct 24 2019 |  |
| 7 | Golden retriever | M | 5 | 33 | 6 | MEAT | July 4 2019 | Aug 1 2019 | Oct 24 2019 |  |
| 8 | Australian shepherd | M | 3 | 21.8 | 5 | PLANT | July 4 2019 | July 30 2019 | DNF | Lost to follow-up |
| 9 | St Bernard x | F | 7 | 50.0 | 6 | Dropped | July 9 2019 | DNF | DNF | Gained excessive weight |
| 10 | German shepherd x collie | F | 7 | 25.5 | 4 | PLANT | July 9 2019 | Aug 15 2019 | Nov 12 2019 |  |
| 11 | German shepherd x collie | F | 4 | 25.0 | 4 | Excluded | July 9 2019 | DNF | DNF | Hypocalcaemia |
| 12 | Labrador retriever | F | 6 | 32.3 | 7 | PLANT | July 11 2019 | Aug 8 2019 | Nov 7 2019 |  |
| 13 | Labrador retriever | M | 4 | 32.5 | 5 | PLANT | July 11 2019 | Aug 8 2019 | Nov 5 2019 |  |
| 14 | Labrador retriever | M | 3 | 27.2 | 5 | PLANT | July 11 2019 | Aug 8 2019 | Nov 5 2019 |  |
| 15 | Akbasha | M | 7 | 45.3 | 6 | PLANT | July 23 2019 | Aug 20 2019 | Nov 21 2019 |  |
| 16 | Collie x | F | 3 | 16.5 | 6 | MEAT | July 23 2019 | Aug 20 2019 | DNF | Lost to follow-up |
| 17 | Klee kai | M | 3 | 8 | 6 | MEAT | Feb 10 2020 | July 7 2020 | Sept 29 2019 | Delay due to COVID |
| 18 | Klee kai | M | 5 | 6 | 5 | MEAT | Feb 10 2020 | July 7 2020 | Sept 29 2019 | Delay due to COVID |
| 19 | Irish wolfhound | F | 4 | 48 | 5 | MEAT | July 23 2019 | Aug 15 2019 | Nov 12 2019 |  |
| 20 | Pug x Dachshund | F | 6 | 10 | 7 | MEAT | July 23 2019 | Aug 20 2019 | Nov 14 2019 |  |
| 21 | Australian shepherd | M | 9 | 17.2 | 4 | PLANT | July 25 2019 | Aug 14 2019 | Nov 20 2019 |  |
| 22 | Poodle | F | 3 | 17.4 | 4 | MEAT | July 25 2019 | Sept 5 2019 | Jan 9 2020 |  |
| 23 | Australian shepherd | F | 6 | 23 | 6 | MEAT | July 25 2019 | Aug 15 2019 | Nov 12 2019 |  |
| 24 | Corgi x | F | 3 | 11.5 | 7 | PLANT | Nov 13 2019 | Dec 18 2019 | DNF | Developed UTI |
| 25 | Husky x labrador retriever | F | 5.5 | 27 | 5 | DNF | March 5 2020 | DNF | DNF | Dropped out due to COVID |
| 26 | Boxer x | M | 2.5 | 35 | 5 | MEAT | Sept 10 2019 | Oct 10 2019 | Mar 18 2020 | Delay due to COVID |
| 27 | Labrador x poodle | F | 2 | 28 | 7 | PLANT | Oct 1 2019 | Oct 29 2019 | Feb 11 2020 |  |
| 28 | Labrador x | M | 2.5 | 31.5 | 6 | MEAT | Nov 13 2019 | Dec 19 2019 | DNF | Dropped out due to COVID |
| 29 | Bichon x | M | 3.5 | 7.6 | 7 | MEAT | Sept 12 2019 | Nov 14 2019 | Feb 13 2020 |  |
| 30 | Golden retriever | M | 5 | 25 | 5 | PLANT | Oct 3 2019 | Oct 31 2019 | Feb 13 2020 |  |
| 31 | Potcake | F | 3 | 14 | 5 | PLANT | Feb 12 2010 | July 6 2020 | Sept 28 2020 |  |
| 32 | Cattledog x collie | M | 8 | 28 | 6 | MEAT | Oct 3 2019 | Nov 7 2019 | Feb 13 2020 |  |
| 33 | Cocker spaniel x miniature poodle | M | 2 | 8.2 | 7 | PLANT | Oct 3 2019 | Oct 31 2019 | July 9 2020 | Delay due to COVID |
| 34 | Soft-coated wheaten terrier | M | 2 | 21.5 | 6 | MEAT | Nov 19 2019 | Dec 18 2019 | Mar 17 2020 |  |
| 35 | Cattledog x collie | M | 9 | 40 | 7 | PLANT | Nov 19 2019 | Jan 9 2020 | Mar 18 2020 |  |
| 36 | French bulldog | M | 7 | 17.2 | 6 | DNF | Nov 20 2019 | DNF | DNF | Dropped out due to COVID |
| 37 | German shepherd x | F | 7 | 22 | 7 | PLANT | July 9 2020 | Aug 4 2020 | Oct 27 2020 |  |
| 38 | Coton du Tulier x miniature poodle | F | 4 | 7.5 | 7 | PLANT | Nov 21 2019 | Dec 18 2020 | Mar 19 2020 |  |
| 39 | Labrador retriever | F | 4.5 | 33 | 7 | MEAT | Nov 21 2019 | Jan 9 2020 | Mar 19 2020 |  |
| 40 | Labrador x | F | 5 | 33.5 | 7 | PLANT | Nov 26 2019 | March 17 2020 | July 8 2020 | Delay due to COVID |
| 41 | Potcake | M | 3 | 18.5 | 4 | PLANT | Nov 26 2019 | March 17 2020 | July 8 2020 | Delay due to COVID |
| 42 | German shorthair pointer | M | 6 | 34 | 4 | DNF | Feb 14 2020 | DNF | DNF | Dropped due to anal gland issues |
| 43 | German shorthair pointer | M | 2 | 27 | 4 | DNF | Feb 14 2020 | DNF | DNF | Dropped due to anal gland issues |
| 44 | German shorthair pointer | F | 2 | 17 | 4 | MEAT | July 13 2020 | August 10 2020 | Nov 27 2020 |  |
| 45 | German shorthair pointer | F | 8 | 30.5 | 6 | MEAT | July 13 2020 | August 10 2020 | Nov 27 2020 |  |
| 46 | Labrador x | F | 2 | 20 | 7 | PLANT | Feb 12 2020 | July 14 2020 | Oct 9 2020 | Delay due to COVID |
| 47 | Border collie | M | 3 | 24 | 7 | PLANT | Feb 12 2020 | July 8 2020 | Sept 30 2020 | Delay due to COVID |
| 48 | Springer spaniel | M | 2 | 17.7 | 5 | DNF | Feb 14 2020 | DNF | DNF | Dropped out due to COVID |
| 49 | Mastiff x | M | 8 | 36.5 | 5 | PLANT | June 30 2020 | Aug 3 2020 | Oct 26 2020 |  |
| 50 | Yorkshire terrier | F | 5 | 5.2 | 5 | MEAT | Feb 14 2020 | July 10 2020 | Oct 29 2020 | Delay due to COVID |
| 51 | Border collie | F | 5 | 17.5 | 5 | MEAT | June 30 2020 | July 30 2020 | Oct 23 2020 |  |
| 52 | Golden retriever x poodle | F | 8 | 28.5 | 7 | MEAT | June 29 2020 | July 31 2020 | Oct 26 2020 |  |
| 53 | Labrador x australian shepherd x cattledog | M | 2 | 30.8 | 6 | MEAT | July 10 2020 | Aug 5 2020 | Nov 5 2020 |  |
| 54 | Labrador | F | 4 | 24 | 6 | DNF | June 30 2020 | DNF | DNF | Dropped out due to COVID |
| 55 | Australian shepherd | F | 2 | 16.5 | 5 | PLANT | June 30 2020 | July 28 2020 | Oct 21 2020 |  |
| 56 | Mix | F | 8 | 20 | 5 | PLANT | July 13 2020 | Aug 27 2020 | Nov 20 2020 |  |
| 57 | Boxer x | F | 9 | 20 | 5 | PLANT | July 13 2020 | Aug 27 2020 | Nov 20 2020 |  |
| 58 | Miniature fox terrier | M | 7 | 5.6 | 5 | PLANT | July 13 2020 | Aug 26 2020 | Nov 29 2020 |  |
| 59 | Anatolian shepherd | M | 2.5 | 40 | 5 | MEAT | Feb 10 2020 | July 6 2020 | Sept 28 2020 | Delay due to COVID |
| 60 | Jack Russel terrier | M | 5 | 9.7 | 7 | PLANT | July 10 2020 | Aug 7 2020 | Nov 6 2020 |  |
| 61 | German shepherd dog | F | 3 | 28 | 4 | PLANT | July 13 2020 | Aug 3 2020 | Nov 3 2020 |  |
| 62 | Shepherd x | M | 5 | 35.5 | 6 | MEAT | June 29 2020 | July 29 2020 | Oct 23 2020 |  |
| 63 | Golden retriever | F | 3 | 27.4 | 6 | PLANT | June 29 2020 | July 31 2020 | Oct 30 2020 |  |
| 64 | Mix | M | 4 | 24.5 | 5 | PLANT | June 29 2020 | July 31 2020 | Oct 23 2020 |  |
| 65 | Springer spaniel | F | 2 | 13.3 | 5 | MEAT | June 29 2020 | July 27 2020 | Oct 19 2020 |  |
| 66 | Australian shepherd | M | 9.5 | 19 | 6 | MEAT | June 30 2020 | July 27 2020 | Oct 19 2020 |  |
| 67 | Australian shepherd | F | 9 | 20.3 | 7 | MEAT | June 30 2020 | July 30 2020 | Oct 22 2020 |  |
| 68 | Nova Scotia Duck Tolling Retriever | F | 9 | 25 | 7 | MEAT | June 30 2020 | July 30 2020 | Oct 22 2020 |  |
| 69 | Irish wolfhound | F | 5.5 | 62 | 7 | PLANT | June 30 2020 | July 28 2020 | DNF | GI ulcers after NSAID administration |
| 70 | Hound x | M | 2 | 25.7 | 5 | PLANT | June 30 2020 | Aug 4 2020 | Oct 26 2020 |  |
| 71 | German shepherd | F | 2 | 27.5 | 4 | PLANT | June 30 2020 | Aug 4 2020 | Oct 26 2020 |  |
| 72 | Cattledog x collie | F | 3 | 33.5 | 7 | PLANT | June 30 2020 | Aug 6 2020 | Oct 28 2020 |  |
| 73 | Miniature schnauzer | F | 6 | 7.3 | 5 | MEAT | June 29 2020 | July 27 2020 | Oct 20 2020 |  |
| 74 | Boxer | M | 3 | 33.8 | 6 | MEAT | July 10 2020 | Aug 7 2020 | Nov 3 2020 |  |
| 75 | Shepherd x | F | 2.5 | 18 | 5 | PLANT | June 29 2020 | Aug 5 2020 | Oct 30 2020 |  |
| 76 | German shepherd | F | 5 | 25.5 | 5 | MEAT | July 9 2020 | Aug 3 2020 | Oct 28 2020 |  |

F = female spayed, M = male castrated, DNF = did not finish, x = cross breed, COVID = global Coronavirus pandemic, NSAID = non-steroidal anti-inflammatory drug

**Table 2. Nutrient profile and ingredient list of the commercial animal-based diet (MEAT) and the experimental plant-based diet (PLANT) fed to client-owned dogs in the diet trial, compared to industry recommendations at the time of manufacture.**

| Nutrient | PLANT | | MEAT | | Adult Maintenance  [1](AAFCO, 2018)[1] |
| --- | --- | --- | --- | --- | --- |
|  | /100kcal | % as fed | /100kcal | % as fed | /100kcal |
| Moisture (g) | 1.62 | 6.80 | 1.66 | 6.82 | No recommendation |
| **Metabolizable energy (kcal/100g) | 419 | 4190 | 410 | 4100 |  |
| Ash (g) | 1.69 | 7.10 | 1.98 | 8.10 |  |
| Crude fibre (g) | 0.93 | 3.90 | 0.83 | 3.40 |  |
| *Nitrogen-free extract (g) | 11.36 | 47.60 | 11.00 | 45.10 |  |
| Crude protein (g) | 5.65 | 23.68 | 6.76 | 27.74 | 4.5 |
| Crude fat (g) | 3.56 | 14.90 | 3.22 | 13.20 | 1.38 |
| Calcium (g) | 0.33 | 1.40 | 0.39 | 1.60 | 0.125 – 0.625 |
| Phosphorus (g) | 0.20 | 0.83 | 0.27 | 1.10 | 0.100 |
| Ca:P | 1.69 | | 1.44 | | 1-2:1 |
| Potassium (g) | 0.19 | 0.78 | 0.19 | 0.78 | 0.15 |
| Sodium (g) | 0.06 | 0.24 | 0.10 | 0.40 | 0.020 |
| Chloride (g) | 0.19 | 0.80 | 0.37 | 1.50 | 0.030 |
| Magnesium (g) | 0.03 | 0.11 | 0.03 | 0.13 | 0.015 |
|  |  | mg/100g |  | mg/100g |  |
| Iron (mg) | 9.31 | 39 | 4.88 | 20 | 1.0 |
| Copper (mg) | 0.43 | 1.8 | 0.54 | 2.2 | 0.183 |
| Manganese (mg) | 0.88 | 3.8 | 0.98 | 4.0 | 0.125 |
| Zinc (mg) | 3.58 | 15.0 | 4.63 | 19.0 | 2.0 |
| Iodine (mg) | 0.05 | 0.2 | 0.10 | 0.4 | 0.025 – 0.275 |
|  |  | IU/100g |  | IU/100g |  |
| Vitamin D2 (IU) | 22.44 | 94 | 0 | 0 | No recommendation |
| Vitamin D3 (IU) | 0 | 0 | 16.34 | 67 | No recommendation |
| Vitamin D (IU) | 22.44 | 94 | 16.34 | 67 | 12.5 - 75.0 |
| **PLANT ingredients** | | | | | |
| Peas, barley, oats, potato protein, sunflower oil (preserved with mixed tocopherols), pea protein, lentils, quinoa, calcium carbonate, dicalcium phosphate, primary dried yeast, flaxseed, natural vegetable flavouring, salt, dried marine algae, choline chloride, vitamins (vitamin A supplement, vitamin D2 supplement, vitamin E supplement, niacin, L-ascorybyl-2-polyphosphate (a source of vitamin C), d-calcium pantothenate, thiamine mononitrate, riboflavin, pyridoxine hydrochloride, folic acid, biotin, vitamin B12 supplement), minerals (zinc proteinate, iron proteinate, copper proteinate, zinc oxide, manganese proteinate, copper sulphate, ferrous sulphate, calcium iodate, manganous oxide, selenium yeast), DL-methionine, potassium chloride, L-lysine, taurine, L-carnitine, dried rosemary | | | | | |
| **MEAT ingredients** | | | | | |
| Chicken meal, de-boned chicken, whole brown rice, white rice, oatmeal, chicken fat (preserved with mixed tocopherols), potatoes, salmon meal, natural chicken flavour, whole dried egg, flaxseed, pea fibre, alfalfa, apples, carrots, cranberries, sodium chloride, potassium chloride, dried chicory root, dried Lactobacillus acidophilus fermentation product, dried Enterococcus faecium fermentation product, vitamins (vitamin A supplement, vitamin D3 supplement, vitamin E supplement, niacin, L-ascorbyl-2-polyphosphate (a source of vitamin C), d-calcium pantothenate, thiamine mononitrate, beta-carotene, riboflavin, pyridoxine hydrochloride, folic acid, biotin, vitamin B12 supplement), minerals (zinc proteinate, iron proteinate, copper proteinate, zinc oxide, manganous proteinate, copper sulphate, ferrous sulphate, calcium iodate, manganous oxide, selenium yeast), DL-methionine, L-lysine, taurine, yucca schidigera extract, dried rosemary. | | | | | |

*Nitrogen free extract is an approximation of carbohydrate content, calculated by subtraction of the sum of crude protein, crude fat, crude fibre, moisture, and ash from a total of 100 [1].

**Metabolizable energy (kcal/kg) is calculated as: 10[(3.5 x crude protein)+(8.5 x crude fat)+(3.5 x nitrogen-free extract) [1].


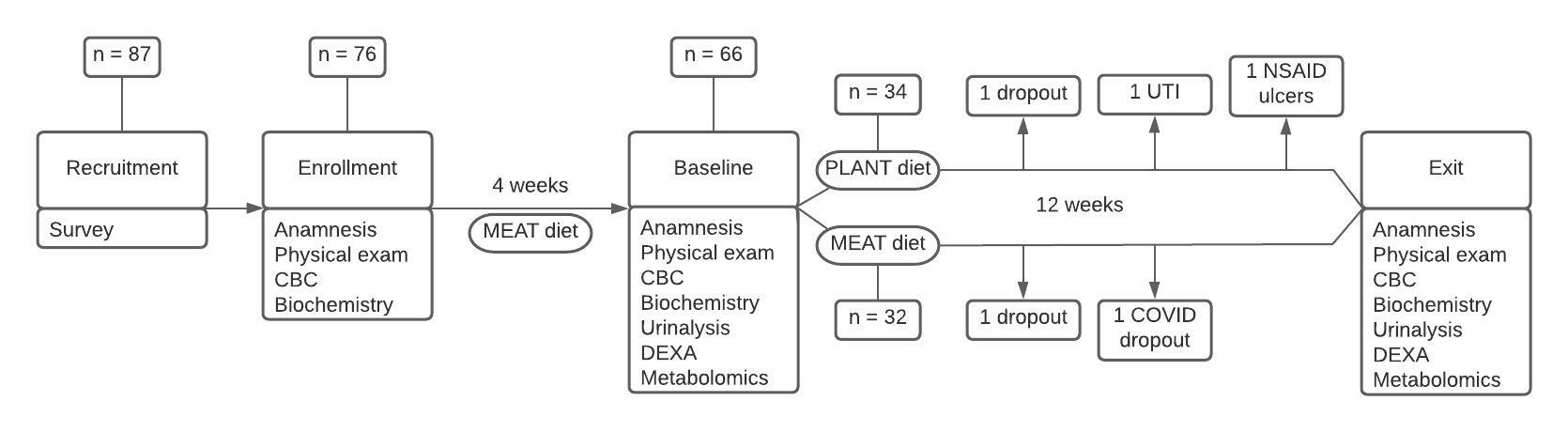


**Figure 1: Diet trial timeline**

Note: the dogs with UTI and NSAID ulcers were removed from the study.

**Table 3. Distribution of independent variables between groups in the diet trial.**

|  | PLANT (n = 34) | | MEAT (n = 32) | |
| --- | --- | --- | --- | --- |
|  | Median | Range | Median | Range |
| Age (years) | 4.0 | 2 – 9.5 | 4.8 | 2 – 9.5 |
| Weight (kg) | 24.9 | 5.4 – 59.1 | 26.6 | 4.8 – 45.7 |
| BCS (1-9) | 5 | 4 - 7 | 6 | 4 – 7 |
|  | Frequency | Proportion | Frequency | Proportion |
| Female | 18 | 43% | 18 | 56% |

**References:**

1. AAFCO, *Official Publication*. 2018, Association of American Feed Control Officials: Champaign, Illinois.
